# Supplementary figures and images for: The Aliment to Bodily Condition knowledgebase (ABCkb): a database connecting plants and human health
Source: BMC Res Notes. 2021 Nov 27;14:433. doi: 10.1186/s13104-021-05835-x (PMC8627056; doi:10.1186/s13104-021-05835-x)

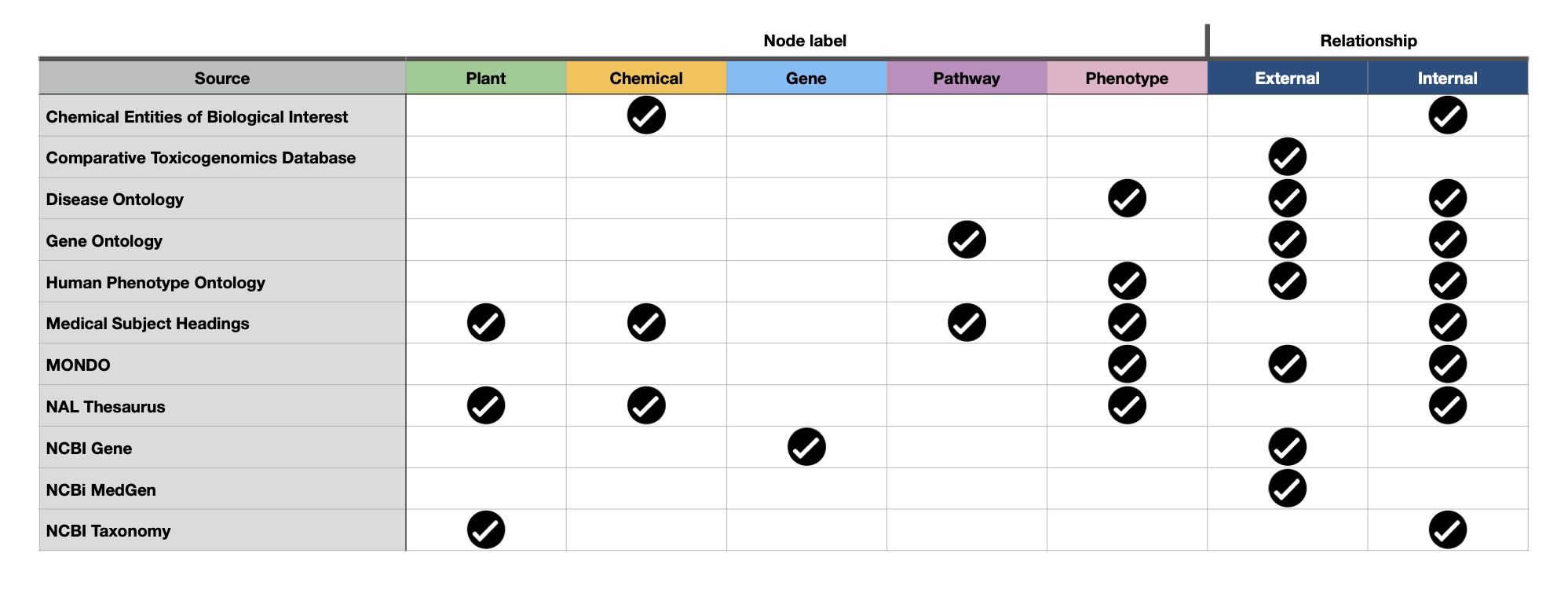

Supplement: Supplementary file 1 — Additional file 1: Figure S1. ABCkb data sources. Data from each source is transformed into one of the 5 labels and may provide external and internal references to nodes within the knowledgebase. The CTD provides manually curated references between labels with no original node labels. [file 13104_2021_5835_MOESM1_ESM.png]

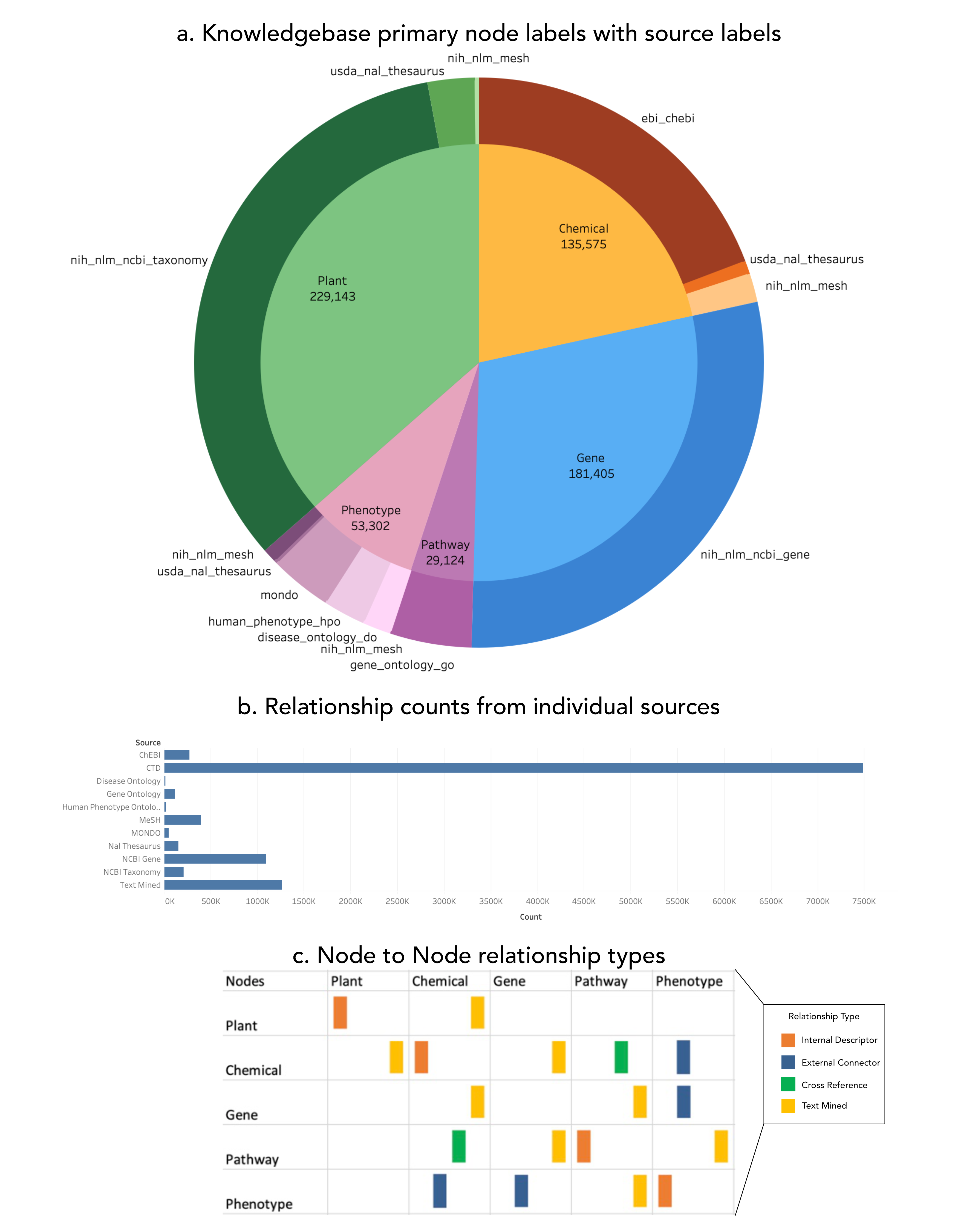

Supplement: Supplementary file 2 — Additional file 2: Figure S2. ABCkb node and relationship statistics. a) The pie chart shows primary labels indicated by color with named secondary (source) labels, shaded and sized by proportion of total nodes in the knowledgebase.b) The sum of relationship counts for each source is indicated by the bar chart. c) Relative relationship counts indicated from node-node in rows, columns in a bar chart in order by type (Internal Descriptor, External Connector, Cross Reference, and Text Mined). [file 13104_2021_5835_MOESM2_ESM.png]
